# An asymmetric model of the spinal locomotor central pattern generator: insights from afferent stimulations

**DOI:** 10.1186/1471-2202-14-S1-P161

**Published:** 2013-07-08

**Authors:** Shelby B Dietz, Natalia A Shevtsova, Ilya A Rybak, Ronald M Harris-Warrick

**Affiliations:** 1 Department of Neurobiology and Behavior, Cornell University, Ithaca, NY, USA; 2 Department of Neurobiology and Anatomy, Drexel University College of Medicine, Philadelphia, PA, USA

## 

We investigated the effects of dorsal root stimulation (flexor related dL2 and extensor related dL5) on fictive locomotion evoked pharmacologically (by 5-HT+NMDA+DA) in the isolated neonatal mouse spinal cord. In our experiments, electrical stimulation produced a wide variety of effects depending on stimulation frequency, intensity, and the drug concentrations used. At stimulation intensities near threshold we were able to produce phase advances and delays that resolved within a single step cycle. During these single-cycle alterations we observed different effects after stimulating dL2 and dL5. Stimulation of dL2 during ipsilateral extension typically produced an early onset of the next flexion and termination of current extension in the ipsilateral activity with or without rhythm disturbances on the contralateral side of the cord (subject to drug concentrations and stimulus intensity). These disturbances represented a true phase resetting characterized by a full flexor phase expression independent of ending the stimulation producing the disturbance. In contrast, stimulation of dL5 often produced a complex bilateral effect starting from a brief activation of ipsilateral extension (for the stimulus duration) with a corresponding reduction in the flexor activity followed by a reactivation of ipsilateral flexor activity (a full second burst that could represent a rebound evoked by end of stimulation) and then by an enhanced next extensor burst. Our results were consistent with the previously proposed two-level architecture of the spinal central pattern generator (CPG) consisting of a top-level rhythm generator (RG) and pattern formation (PF) circuits [1,3]. The above differences in the effects of flexor and extensor afferent stimulation are consistent with the previous suggestion that the spinal CPG has an asymmetric flexor-extensor organization, so that only the flexor half-center of the rhythm generator (RG) has intrinsic rhythmogenic capabilities [2]. To further evaluate the organization of the spinal CPG and its control by flexor and extensor afferents, we extended our previous model of the CPG in the neonatal rodent spinal cord [4] by incorporating bilateral flexor and extensor afferent pathways and used this model for simulating the above effects of afferent stimulations. The extended model contains left and right half-center RGs interacting via excitatory and inhibitory commissural interneurons (CIN). The flexor half-center of each RG can intrinsically generate rhythmic bursting, while the extensor half-centers do not have intrinsic rhythmic capabilities. Each RG half-center projects to the corresponding PF population controlling the activity of the corresponding motoneuron pools. Ipsilateral neural circuits include several populations of spinal interneurons, including experimentally identified CINs, two types of V2a interneurons, and motoneurons [4]. Network interactions have been organized to be consistent with the activity of these neurons observed during spontaneous experimental deletions [4]. The model reproduces the locomotor-like rhythm with bilaterally coordinated flexor and extensor activities and the neuronal firing patterns observed in the normal conditions and during resetting and non-resetting deletions [4], as well as the experimentally evoked effects of dorsal root stimulation described above. The model proposes mechanistic explanations for the asymmetric effect of afferent stimulation and provides novel insights into the organization of the locomotor central pattern generator.
